# Comprehensive Review of How Endoscope Channel Biofilm Differs from Hydrated and Dry Surface Biofilm and Its Implications

**DOI:** 10.1055/a-2965-6797

**Published:** 2026-09-30

**Authors:** Michelle J. Alfa

**Affiliations:** 1 MicrobiologyAlfaMed Consulting Ltd.WinnipegCanada

**Keywords:** quality and logistical aspects, hygiene, performance and complications, endoscopy upper GI tract, endoscopy lower GI tract, pancreatobiliary (ERCP/PTCD)

## Abstract

**Background and Study Aims** Flexible endoscope-related infection outbreaks with respect to the biofilm in endoscope channels continue to be reported. This article reviews how endoscope fixed-biofilm accumulation (EFBA) develops in flexible endoscope channels over repeated clinical use. The aim of this review is to highlight how EFBA differs from dry surface biofilm (DSB) and hydrated biofilm (HB).

**Patients/Materials and Methods** The process of (EFBA) formation in patient-used endoscope channels is compared to that of DSB and HB. The inadequate (or lack) of cleaning in narrow endoscope channels and the impact of high level disinfection (HLD) fixing of the EBFA within the endoscope channels result in a very different matrix compared to DSB or HB. The impact of fixed EFBA on microorganism survival within the matrix provides a more stringent and realistic challenge compared to DSB or HB. The choice of an appropriate multispecies biofilm model is also crucial to ensuring that it closely mirrors the clinical aspects related to EFBA.

**Results** This comprehensive review confirms that EFBA is a driver of persistent endoscope channel contamination and exogenous transfer of pathogens to patients that can lead to infection. The literature review demonstrates that mono-microbic HB is the most common biofilm model used over the past 20 years, whereas models that integrate rounds of fixation such as the endoscope cyclic buildup biofilm (ECBB) and endoscope buildup biofilm (EBBF) better reflect the clinical aspects of EFBA in patient-used endoscope channels.

**Conclusions** The published data support the need for the use of an appropriate fixed-biofilm model when validating cleaning and disinfection processes for flexible endoscope channels, thereby ensuring an optimal margin of safety during clinical use.

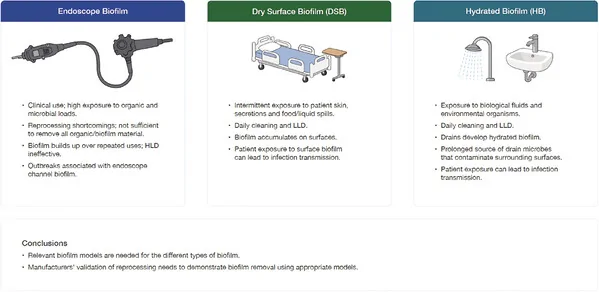

## Introduction

The need for high level disinfection (HLD) or low temperature sterilization for reducing the risk of contaminating microorganisms in fully reprocessed endoscopes has been recognized since 1974. 1 Furthermore, the importance of drying channels to prevent bacterial replication during endoscope storage of patient-used, fully reprocessed endoscopes was reported as early as 1991. 2 Over the years, since these initial publications, the problem of endoscope channel contamination due to bacterial replication and biofilm formation within fully reprocessed flexible endoscope channels continues to the present times. 3 4 5 6 7 8 9 10 11 12 13 14 15 16 17 18 19 20 21 22 23 24 25 26 27 28 29 30 31 32 33 34 35 36 37 38 39 As outlined by Rowen et al., “Disinfection and sterilization processes can fail if medical device cleaning has not been conducted appropriately, including if they promote fixing of Biofilms to surfaces.” 40 Furthermore, Arvand et al. state that “It is noteworthy that the currently available and used international standards for disinfectant efficacy testing do not cover the biofilm phenotype.” 41 This is an important shortcoming as recent outbreaks of multiresistant *Klebsiella pneumoniae* and *Pseudomonas aeruginosa* have implicated wet channels that allow biofilm formation as the underlying problem leading to endoscope-associated outbreaks. 42 43 44 It has been shown that even the American Type Culture Collection (ATCC) reference strains of *K. pneumonia* (non carbapenemase producing) embedded in biofilm (as opposed to planktonic organisms) required increased levels of peractic acid (PAA) to provide a 5 Log _10_ reduction *.* 41 42 Furthermore, outbreak strains of carbapenemase-producing *K. pneumonia* within the biofilm show tolerance to PAA. 41 42 45 In addition, Cholley et al. demonstrated that *K. pneumoniae* in hydrated biofilm (HB) could survive exposure to 2% glutaraldehyde (GLUT) and that prior desiccation increased the survival rate. 45

The recent review by Ofstead et al. reported that failure of manual cleaning (21/26) and failure of drying/storage (12/26) are common reprocessing breaches in clinical practice and that from 2019 to 2024, numerous outbreaks linked to flexible endoscopes have occurred. 3 They concluded that the published data “…showed that HLD was not reliably effective.” Furthermore, they recommended that “Optimizing pre-treatment, processing timeliness, and manual cleaning will help prevent soil accumulation and biofilm formation.” 3 The published data to date confirm that pathogens (antibiotic resistant organisms (AROs) as well as non-AROs) embedded in biofilm that forms within the endoscope channels is due to an ongoing reprocessing issue. There is an urgent need to provide a realistic endoscope channel biofilm model that could be used to evaluate any future technology developments aimed at reducing the risk of biofilm formation within endoscope channels.

The first objective of this review is to better understand how biofilm in endoscope channels develops and accumulates over repeated use and how it differs from DSB found on healthcare surfaces and also differs from the traditional HB found in drains of sinks and showers in the health care setting. The adverse impact of EFBA highlights the shortcomings of the current reprocessing validation requirements for endoscope manufacturers. The second objective is to provide insight into an optimal experimental biofilm model to investigate how improvements in flexible endoscope cleaning, disinfection/sterilization, and dry endoscope storage can reduce/eliminate endoscope biofilm. Finally, the impact of EFBA on the transmission of clinical infections will be reviewed.

## How Does Biofilm Form in Endoscope Channels?

Each time a flexible endoscope is used for a patient procedure, the exterior surface of the endoscope that is inserted into the patient as well as the entire interior surface of endoscope air/water and instrument channels (as well as any other channels used) is exposed to high levels of human organic material (e.g., mucosal secretions, feces, urine, blood, etc.) and microorganisms present in the gastrointestinal, genitourinary, or respiratory tract (e.g., *Escherichia coli, Klebsiella pneumonia, Enterococci species, yeast,* etc.) or the upper respiratory tract (e.g., *Streptococcus species, Staphylococcus species,* etc.). 5 46 47 This high organic and high microbial exposure is especially true for invasive duodenoscope endoscopic retrograde cholangiopancreatography (ERCP) procedures and for unprepped urgent gastrointestinal bleeds. 5 The historical expectation was that the organic and microbial contaminants acquired during the clinical procedure would be substantially reduced by bedside flushing followed by manual cleaning and/or automated cleaning using detergent. 48 The subsequent exposure of endoscope channels to HLD or low temperature sterilization was expected to eliminate any low levels of remaining microorganisms (except for low levels of spores after HLD). 48 The current manufacturer validation of manual and automated flexible endoscope-cleaning protocols requires a representative fluid organic soiling challenge but not a microbial challenge, 49 as the subsequent HLD treatment was expected to eliminate any low level of microorganisms (except low levels of spores).

As reviewed by Mishra, an “initial conditioning film” is an essential first requirement for any type of biofilm formation. 6 It is likely that inadequate cleaning of new flexible endoscope channels after initial patient use results in very low levels of organic residuals within the endoscope channels that are subsequently “fixed” by exposure to HLDs and/or sterilant, creating an initial conditioning film. The inadequate cleaning could be due to shortened duration of manual cleaning 7 or due to narrow channels (e.g., air/water channels) that have detergent flow but no friction (i.e., no brushing) during the manual cleaning stage. 50 Each repeated clinical use would contribute to accumulation of this initial conditioning film. This initial conditioning film of organic residuals would then provide a nutrient source that facilitates the attachment of human and/or environmental organisms that replicate within the organic layered matrix to form the initial biofilm. With repeated clinical exposures, mature EFBA would develop. Subsequently, gut organisms of high concern can become embedded and replicate within the initial and/or mature biofilm.

Endoscopes are exposed repeatedly to high levels of microorganisms (between 6 to 8 Log _10_ per endoscope suction channel) during clinical use. 46 In addition, they also have repeated exposure to the final Automated Endoscope Reprocesser (AER) or manual rinse water that may contain microbial contaminants 5 51 52 as well as exposure to environmental organisms (including spore-forming *Bacillus* spp.) during storage that could result in environmental organisms attaching to the initial conditioning film or initial/mature biofilm. The postmarket clinical study by Okamoto et al. reported that out of 264 clinically used and reprocessed duodenoscopes sampled, environmental organisms were detected in 48.4% of the instrument channels ( *Bacillus* spp. were the most isolated organism representing 61.1% of 432 environmental organisms detected). 47 *Bacillus* spp. could be present in their spore form within endoscope channels, which would protect them from HLD. Recent studies from many different countries confirm that fully reprocessed flexible endoscopes often still have moisture in the channels due to inadequate drying. 3 42 53 54 55 56 57 58 59 This moisture during storage allows these environmental organisms (e.g., low concern organisms such as *Bacillus species,* etc.) to integrate into the conditioning biofilm and replicate to form the “initial” biofilm, which over repeated clinical use becomes EFBA. This is most pronounced for HLDs that promote “fixing of biofilms.” 40 High concern organisms (HCOs) such as gut organisms could then become embedded in the EFBA matrix and be protected from subsequent HLD and/or sterilization. Such embedded Gram-negative microbes can get “by-stander protection” from HLDs and/or antibiotics that are offered by the environmental organisms present in the initial biofilm matrix. 60 The enhanced complexity of the “mixed species” biofilm matrix makes it even harder to eliminate it when compared to the mono-species biofilm. 56 61 As reported by Vestweber et al., the 72-h dual-species traditional biofilm consisting of *P. aeruginosa* and *S. aureus* had significantly reduced antibiotic penetration, which resulted in a 50% increase in tolerance to gentamicin. 62 They concluded that their data underscored the importance of using a multispecies biofilm to more comprehensively understand how microbial communities impact the effect of antibiotic treatments. 62 This would also be true for HLD and low-temperature sterilization of endoscope channels containing multispecies EFBA. Tan et al. reported that a culture of the endoscope channels done confirmed not only the presence of the outbreak strain of *P. aeruginosa* but also reported that there were other organisms coisolated, indicating that a polymicrobic biofilm does form in patient-used endoscope channels. 56 Okamoto et al. also demonstrated the wide range of multiple low/moderate organisms that were detected together on cultures of clinically used, fully reprocessed duodenoscope instrument channels. 47

The formation of EFBA would be expected to develop faster in narrow endoscope channels (e.g., Air/water channels) that cannot be brushed as there is no friction used during many of the current manufacturer’s validated cleaning processes. Moshkanbaryans et al. used the endoscope cyclic buildup biofilm (ECBB) model and demonstrated that without friction in these narrow channels, the current manufacturer’s cleaning instructions cannot remove ECBB. 50 Primo et al. reported that EFBA in the insertion tube portion of newly replaced Air/water channels of clinically used gastroscopes was rapidly re-established from the umbilical portion of the channels when only the insertion tube portion of the channels was replaced during repairs. 57 In addition, Northage et al. reported that culture methods may not detect very low levels of viable microorganisms after HLD in mixed species traditional biofilm. 63 Furthermore, viable but non-culturable (VBNC) organisms remaining in channels can survive HLD and regrow to high levels after 24 h in broth. 63

EFBA that accumulates in channels over repeated clinical uses creates significant challenges to the efficacy of manual and automated cleaning as well as to the efficacy of HLD and/or sterilization. To address this challenge, it would be important to integrate multi-species EFBA models in the required testing for manufacturer’s instructions for cleaning and HLD/Sterilization protocols for flexible endoscopes.

## Why is the Current Validation Process for Flexible Endoscopes Insufficient?

The current manufacturer validation of microbial elimination by HLD in flexible endoscopes has only been evaluated using a worst-case planktonic microorganism challenge in the presence of a representative organic challenge followed by drying that must show a 5 Log _10_ microbial reduction. 64 However, there was an initial lack of recognition that after repeated rounds of clinical use, reprocessing and storage that fixed biofilm could accumulate within endoscope channels and this would be far more difficult to remove during manual or automated cleaning and HLD (or low temperature sterilization) compared to a dried organic fluid challenge containing planktonic microorganisms. The persistent organisms reported as contaminants within endoscope channels after full reprocessing include multi-drug resistant organisms (MDRO). 3 47 However, many other low/moderate concern microbes can also survive in endoscope channels after full reprocessing. This is supported by the Okamoto study that showed there were still positive cultures of Low to Moderate concern organisms (25/25 tested) from the instrument channels of clinically used duodenoscopes that were fully reprocessed and sterilized using ethylene oxide. 47

The recent review by Ofstead et al. reported that Medical Device Reports for flexible endoscopes and AERs submitted to the Food and Drug Association (FDA) have continued to increase from 2019 to 2024. 3 In addition, cultures of fully reprocessed duodenoscopes show that detection of High Concern Organisms (HCO) continues to occur. 3

Alfa reviewed the multiple stages of flexible endoscope reprocessing indicating that “breaches” in any one of these stages could contribute to persistent microbes and organic matter that could lead to biofilm formation within endoscope channels. 65 This is further substantiated by van der Ploeg et al.’s clinical study showing that there was a significantly higher detection rate of HCOs from clinically used duodenoscopes when the manual cleaning phase was less than 5 min (this is substantially less cleaning time than recommended by the manufacturer’s instruction for use (MIFU) which takes about at least 20 min). 7 Ofstead et al. also stated: “ *Although no one study was generalizable due to the diverse characteristics of their facilities and nature of breaches and patient exposures, these heterogenous studies consistently showed that HLD was not reliably effective* ”. 3 Furthermore, the need for improvements in MIFUs was highlighted. The clinical breaches in following the MIFU that have been reported by Ofstead et al. may account for some of the accumulation of EFBA. 3 However, to provide an adequate margin of safety, it is crucial that the MIFU integrates EFBA removal as part of the MIFU reprocessing validation.

## How does EFBA Differ from DSB and HB?

Fig. 1 shows how EFBA differs in many aspects from DSB and HB. EFBA accumulates after repeated clinical use in patients’ body cavities, followed by cleaning and HLD and/or sterilization. DSB accumulates on healthcare surfaces after patient/staff exposure and repeated daily cleaning and low-level disinfection (LLD). HB is biofilm that forms in a continually hydrated environment such as sink and shower drains, as well as in wastewater pipes. All forms of established biofilm (i.e., 72 h formation or greater) such as EFBA, DSB and HB are very difficult to eliminate. 6 41 47 62 66 67 68 69 70 Beaugelin et al. compared three biofilm models and clearly demonstrated that 120 h HB formation in PTFE channels was far more resistant to PAA disinfection compared to even 96 h HB formation in polytetrafluoroethylene (PTFE) channels. 67 Cleaver and Garnett reviewed the current advancements in clinical biofilm research using static growth or dynamic growth biofilm models at 30–37 °C. 61 However, there was no discussion of modelling endoscope biofilm in endoscope channels that develops at room temperature during transport after clinical use to the reprocessing area and during storage at room temperature after full reprocessing.

**Fig. 1:**
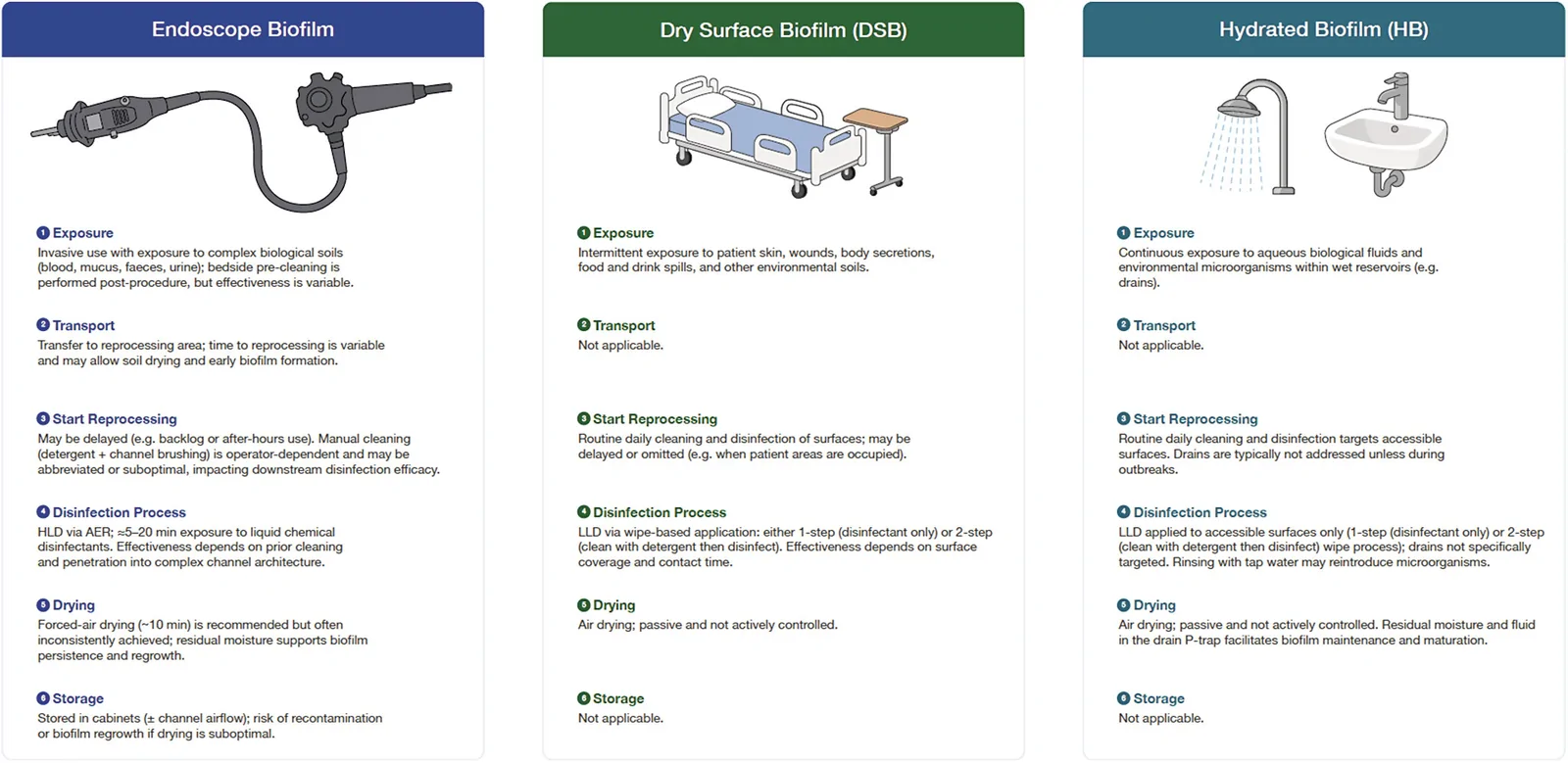
Biofilm formation in the health care setting. 2 7 41 68 Refer to Table 2 for other relevant references. HLD; high level disinfectant, AER; automated endoscope reprocessor, LLD; low level disinfectant.

The fixation provided by HLD and/or sterilization processes creates a much stronger cross-linked matrix in EFBA than that of DSB on healthcare surfaces that is formed by repeated rounds of; cleaning, low level disinfection, and drying. The accumulated mixed species biofilm matrix that is fixed in flexible endoscope channels (especially in narrow channels where there is no friction during the cleaning stage) is also much more difficult to eliminate compared to HB formed on the same substrate. 71 HB that forms within healthcare sink/shower drains rarely gets disturbed or removed unless addressed as part of remedial actions during an ongoing infection outbreak. As such the sheer volume of HB that accumulates undisturbed in healthcare sink, shower and sluice drains over prolonged periods of time makes it especially hard to eliminate ( Fig. 1 ). It is important to recognize that friction is critical to physically disperse and remove all forms of biofilm to ensure efficacy of HLD, sterilization and/or Low level disinfection (LLD). Friction (manual wiping with cloths or wipes) is present for healthcare surfaces that get cleaned/disinfected daily but is absent in cleaning of narrow endoscope channels that cannot be brushed as well as in patient sink and shower drains and wastewater pipes.

Reprocessing of flexible endoscopes should provide the opportunity for better control of EFBA formation as these medical devices are reprocessed after each clinical use whereas healthcare surfaces, sink and shower surfaces are cleaned and disinfected daily and the plumbing of sinks, showers, sluices etc. in health care are infrequently cleaned and disinfected unless there is an outbreak linked to plumbing. If optimum endoscope channel cleaning that incorporates friction is used from the very first clinical use onwards, there is hope that EFBA formation could be prevented and that existing EFBA could be physically removed.

There are newer technology developments for cleaning of endoscope channels that do generate friction in all endoscope channels during the cleaning process. 49 72 Moshkanbaryans et al. 50 used the Endoscope Cyclic Buildup Biofilm (ECBB) 73 model and demonstrated there was excellent physical removal of both fixed organic and microbial components compared to the current manufacturer’s recommended manual cleaning protocols. Nickel et al. used a novel iron oxide particle solution in the cleaning process, showing that it removed traditional HB due to the friction created. 72

## Current Biofilm Models

Fig. 2 shows the cross-section characteristics of DSB, HB, ECBB, and endoscope build-up biofilm (EBBF) models that have been used in research studies. As suggested by Arvand et al. and Beaugelin et al., there is a need for an appropriate biofilm model relevant to the reprocessing of flexible endoscope channels. This model could be used in the manufacturer’s validation of the cleaning instructions meant for flexible endoscope channels as well as for manufacturer’s validation of effective HLD for flexible endoscope channels. 41 67 The model selected should reflect the most difficult-to-clean aspect of the medical device/surface, which would be endoscope PTFE channels. In addition, the model should mimic the repeated rounds of exposure to high organic and microbial loads during clinical use and the type of HLDs that endoscope channels are exposed to after the cleaning phase in clinical use. Finally, the model should reflect the repeated cyclic uses and room temperature storage conditions relevant to clinically used flexible endoscopes.

**Fig. 2:**
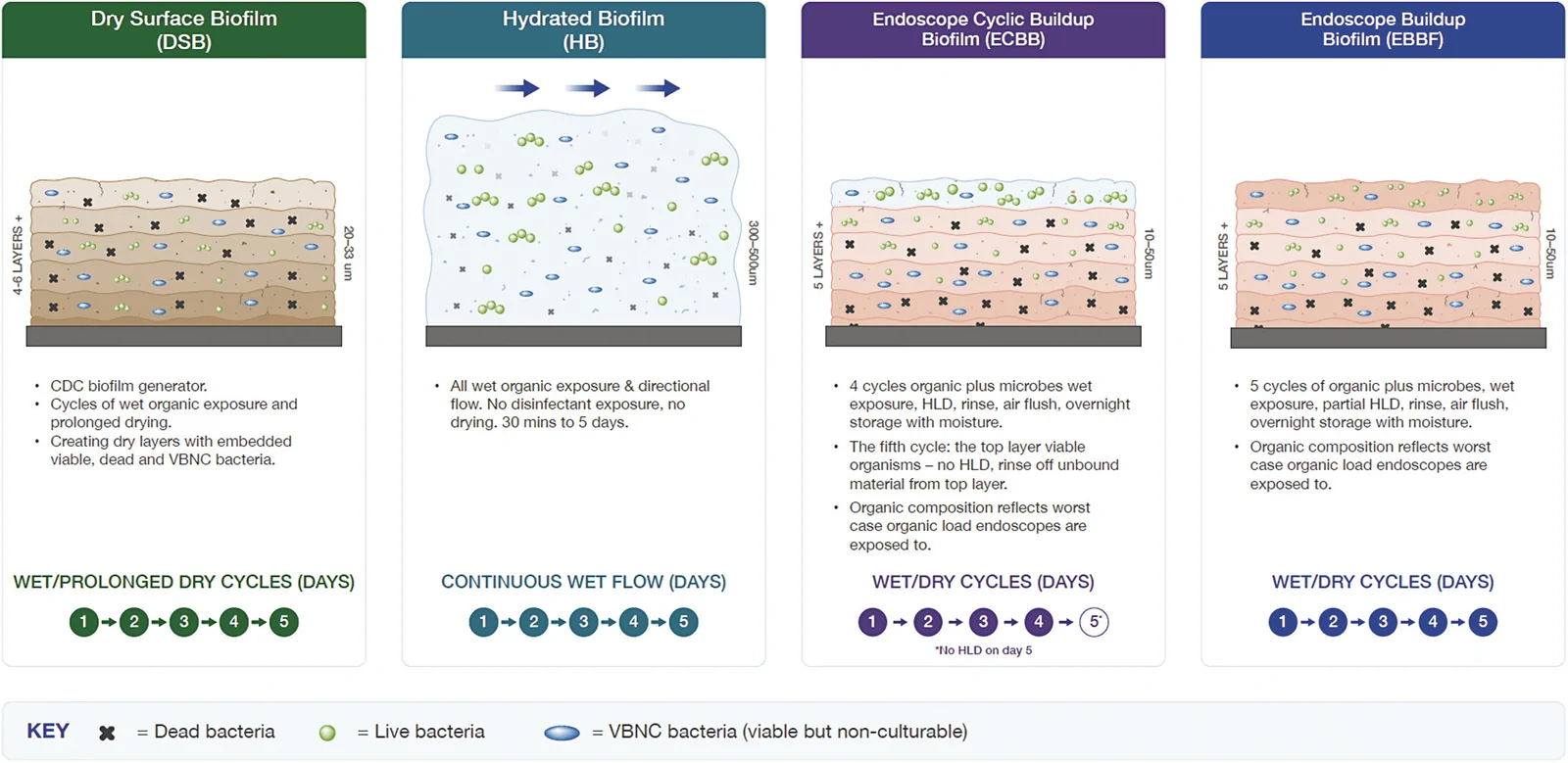
Current health care-related biofilm models. 68 104 Refer to Table 2 for other relevant references. CDC; Center for Disease Control and Prevention, VBNC; viable but non-culturable, HLD; high level disinfection.

Table 1 outlines the diverse types of biofilm models used over the past 20 years to study endoscope cleaning and/or HLD efficacy. Traditional HB models are the predominant format (25/35 publications) that have been used in current years to study cleaning and/or disinfection of flexible endoscope channels. These models include immature HB formed over 24–48 h, as well as mature HB formed over 72 h or more. These published models also span several different formats and surfaces including channels of PTFE, Tygon tubing, polyvinylchloride (PVC) tubing, plastic pegs and plates, as well as microfluidic and glass bead formats.Each of these published articles has been helpful to address various aspects of the impact of biofilm formation and/or accumulation on endoscope reprocessing. However, as indicated by Cleaver and Garnett, “In vitro models for biofilm investigation can be highly tailored to the system that is being modeled.” 61 It is very difficult to extrapolate how some of the published models represent what happens relevant to biofilm in clinically used reprocessed flexible endoscopes (e.g., use of substrates such as various types of non-PTFE tubing, plastic pegs/plates, and glass beads). Often, the immature biofilm ( < 24 h formation) is evaluated, 41 42 63 which is far easier to remove from the substrate it is formed on; also it is easier to kill the organisms within the matrix than in the mature biofilm ( > 72 h). In addition, the substrate used (e.g., glass beads and hard plastic surfaces) may not reflect the clinical PTFE substrate relevant to the adhesion characteristics of the biofilm in terms of ease of physical extraction or HLD penetration.

**Table 1:** Biofilm models used for flexible endoscope research.

Model	Substrate	Organism(s) tested	Microorganisms tested as Poly or Monomicrobic
Hydrated Biofilm (HB): *N* = 25	PTFE *N* = 14	*P. aeruginosa, x3,* 4 67 78 *K. pneumonia,* 45 *[P. aeruginosa, S. aureus]* 37 *E. faecalis* 82 *E. coli x4* 83 84 85 86 *[P. aeruginosa, S. aureus, M. abcessus]* 87 *[P. aeruginosa, S. aureus, E. coli, K. pneumonia]* 66 *[P. aerugiosa, S. epidermidis, E. cloacae, K. pneumonia]* 88	Monomicrobic: 13 publications*
		*{P. aeruginosa & S. aureus & S. epidermidis, & E. coli}* 63	Polymicrobic: 1 publication**
	Plastic wells/plates *N* = 6	*P. aeruginosa x 3,* 89 90 91 *MRSA,* 72 *S. aureus,* 92 *[C. albicans, C. parapsilosis, P. aeruginosa, S. maltophilia]* 93	Monomicrobic: 6 publications*
	Silicone surface *N* = 1	*P. aeruginosa* 94	Monomicrobic: 1 publication
	Tygon Tubing *N* = 1	*K. pneumoniae* 95	Monomicrobic: 1 publication
	Bead Assay* *N* = 1	*K. pneumoniae* 41	Monomicrobic: 1 publication
	PVC Tubing *N* = 1	*[S. aureus, P. aeruginosa]* 96	Monomicrobic: 1 publication*
	MBEC pegs *N* = 1	*{P. aeruginosa & E. faecalis}* 97	Polymicrobic: 1 publication
Buildup Biofilm: (ECBB & EBBF) *N* = 8	PTFE *N* = 6	*{P. aeruginosa & E. faecalis} 5* 50 74 75 98 99 *P. aeruginosa* 100	Polymicrobic: 5 publications** Monomicrobic 1 publication
	Microfluidic *N* = 1	*P. aeruginosa* 76	Monomicrobic: 1 publication
	MBEC *N* = 1	*[P. aeruginosa, E. faecalis, C. albicans]* 101	Monomicrobic: 1 publication*
HB & EBBF *N* = 2	MBEC *N* = 1	*{P. aeruginosa & E. faecalis}* 102	Polymicrobic: 1 publication**
	PTFE *N* = 1	*{P. aeruginosa & E. faecalis}* 73	Polymicrobic: 1 publication**
Total: 35			

HB; hydrated biofilm, ECBB; endoscope cyclic buildup biofilm, 73 EBBF; endoscope buildup biofilm, 74 *[ ] Each microorganism was tested separately. ** { } All microorganisms were tested together.

The HB models grown at 37 °C used for biofilm studies do not reflect the cyclic reprocessing and storage aspects at room temperature, which the clinically used flexible endoscopes are subjected to. Theoretically, endoscope channels should not contain HB, but recent studies have found residual moisture in endoscope channels and have linked this finding to biofilm formation. 41 42 Indeed, Haak et al. reported that despite 6 months of storage after a clinical outbreak related to a multiresistant strain of *P. aeruginosa,* they found that the patient-used endoscope channel still contained residual moisture and was culture-positive for the same outbreak strain. 42 Certainly, moisture in endoscope channels that are stored at room temperature facilitates replication of any viable organisms remaining in these channels, 46 but it does not account for the strength of the accumulated build-up of the fixed material over repeated use. Despite this limitation, there are valuable data obtained from the HB publications that use the glass bead assay. They show the increased resistance to PAA shown by outbreak strains of carbapenemase-producing *K. pneumoniae* as well as by non-carbapenemase-producing *K. pneumoniae* strains. 41 42 The increased tolerance of PAA was not specific to the antibiotic resistance, but rather related to the HB formation. Likewise, the 72 h HB channel model 66 facilitated the study of the effects of thermal plasma on HB formed by four individually tested bacteria. Beaugelin et al. used the HB model in PTFE channels to document as to how the biofilm formed by *P. aeruginosa* after 120 h was far more resistant to PAA than the 90 h biofilm formation. 67

DSB has been used to study the survival of microbes in “dry” health care environments. 68 69 DSB is formed by a cyclic process that includes rounds of hydration and rounds of dry storage at room temperature. This cyclic aspect of DSB reflects the cyclic aspect that is used in reprocessed endoscopes. However, there is no integration of surface disinfection in the DSB model, whereas this is a crucial factor in the ECBB model and the EBBF models. The DSB model has provided a research model that is relevant to surfaces present in health care but may not be as relevant to the type of EFBA that develops in endoscope channels after repeated rounds of clinical use, cleaning, HLD, and/or sterilization and nonsterile storage at room temperature.

As indicated by Cleaver and Garnett, testing a monomicrobic biofilm has been very beneficial but they go on to state: “However, it has been shown that growth as a single-species is not the preferred way that bacteria thrive and survive in natural biofilms…”. 61 Johani et al. extracted and evaluated patient-used endoscope channels using quantitative real-time PCR to determine what microbial residuals were present in the channel biofilm. 52 They found that the air/water channels had higher colony forming unit (CFU)/cm tendencies compared to the instrument channels. 52 They also assessed the microbiome detected in the gastroscope and colonoscope channels and stated: “The presence of these organisms suggests that endoscopes become contaminated with both environmental and patient organisms and these organisms act together to form a strong biofilm that is tolerant to decontamination procedures.” 52

Indeed, the clinical studies that assessed microbial contaminants have demonstrated the polymicrobic nature of residual organisms within fully reprocessed clinical endoscope channels. 47 70 Of the 48.4% of 264 duodenoscopes that grew environmental organisms, *Bacillus species* predominated (264/432) as environmental cohabitants of endoscope channels. 47

Table 1 summarizes the current biofilm models that have been utilized to evaluate endoscope-related biofilms over the past 20 years. Of the 35 biofilm models, the majority (23/35) used bacterial culture broth media as the organic challenge, and biofilm was formed using monomicrobic bacteria (25/35). In addition, only 10/35 integrated cyclic exposure to HLD and room temperature conditions.

Table 2 summarizes the components of the endoscope channel biofilm that should ideally be integrated into models used to study this type of clinical biofilm. Both the ECBB 73 and the EBBF 74 75 76 are relevant to the multispecies fixed-biofilm that accumulates in clinically used flexible endoscope channels. They mirror the cyclic reprocessing of endoscope channels, exposure to both Gram positive and Gram negative gut bacteria and relevant organic residues, the impacts of suboptimal cleaning followed by HLD on the accumulation of material and microbes in endoscope channels, as well as storage overnight under moist conditions at room temperature.

**Table 2:** Current biofilm models.

	Characteristics of current biofilm models			
Ideal model components	ECBB 50 73	EBBF 74 75	HB 4 42 45 66 83 84 85 87 88 103	DSB 68 69
PTFE channel	**√**	**√**	**√**	–
Organic challenge	**√**	**√**	±	±
Multispecies relevant microorganism challenge	**√**	**√**	±	±
HLD (full or partial strength ^1^ )	**√** [full]	**√** [partial]	**–**	NA
Room temperature	**√**	**√**	**–**	**√**
Repeated cycles (at least 5): Each cycle with organic ^2^ and multispecies microorganism challenge ^3^ , HLD, water rinse	**√** [Last cycle no HLD]	**√**	–	±
Hold O/N at RT for each cycle	**√**	**√**	±	**√**
Final water rinse to remove unbound organisms	**√**	**√**	**√**	–
Wipe exterior of channel with alcohol prior to testing	**√**	**√**	NA	NA
Destructive testing to quantify organic residues ^2^ and CFU of each organism	**√**	**√**	**√**	**√**

ECBB: Endoscope cyclic buildup biofilm, EBBF: Endoscope buildup biofilm, HB: Hydrated Biofilm, DSB: Dry surface biofilm.

^1^ Full strength; 2.5% Glutaraldehyde, Partial strength; 1.25% Glutaraldehyde.

^2^ Organic residues: at least one of: protein, TOC, carbohydrate, hemoglobin at concentrations reflective of patient secretions aspirated through endoscope channels.

^3^ Relevant Gram (+) and Gram (−) gut bacteria as well as relevant environmental organisms.

√; is part of model, –; is not part of model, ±; partially met, NA: Not applicable.

Note: Microfluidic EBBF model uses tiny microfluidic channels and not endoscope PTFE channels.

The HB model has been the predominant model used to study endoscope channel EFBA, whereas the DSB model had been the predominant model used to study healthcare DSB. The monomicrobic HB model may be relevant to the initial stages of EFBA in endoscope channels but does not truly reflect the multispecies aspect or the accumulation and fixation aspects of endoscope reprocessing over time. As such, it is not a model that provides a “worst-case” challenge when evaluating cleaning efficacy or HLD/low temperature sterilization efficacy.

A relevant biofilm model is crucial to ensure that the manufacturer’s cleaning and HLD/sterilization protocols can eliminate this great stringent challenge. The manufacturer validation of an AER requires a channel HB challenge. 64 However, many of the currently used flexible endoscopes already contain fixed layers of EFBA, which is much more difficult to remove than HB. Utilizing a more stringent biofilm challenge such as ECBB, or EBBF instead of HB would help ensure that the worst-case challenge that exists within many of the currently clinically used endoscopes is met and would help ensure that new endoscopes do not develop EFBA.

Currently, there have been few studies for evaluating the use of smart bioactive coatings with biofilm-disruptive properties (i.e., antifouling agents) that can help prevent initial biofilm from forming within endoscope channels. 40 In addition to the use of antifouling agents, there needs to be more novel ways to ensure adequate friction is used to remove the initial and/or established EBA. It is this combination of the prevention of the initial formation of EFBA and the effective removal of the established EFBA that currently remains an unmet need in endoscope channel reprocessing.

## Impact of Endoscope Channel EFBA on Infection Control

Endoscope-related infections and/or outbreaks are still occurring. 3 4 5 6 11 20 22 33 42 43 56 77 78 Cisse et al. clearly demonstrated that it is not just MDROs being implicated in outbreaks but that non-MDRO *P. aeruginosa* can also cause endoscope-associated “silent” outbreaks. 77 They identified that both the AER contamination with this strain and the biofilm persistence within the duodenoscope channels played a role in the clinical outbreak. 77 The use of whole genome sequencing (WGS) was “…pivotal in distinguishing transient contamination from true persistent transmission.” 77

Persistence of MDROs in contaminated endoscope channels despite culture monitoring (i.e., false-negative cultures) has been documented and can result in continued transmission of MDROs to subsequent patients. 4 77 This may be related to VBNC forms embedded within the biofilm matrix. 51 75 77 van der Ploeg et al. and Cisse et al. have documented that after HLD, the cultures of clinically used endoscope channels may be falsely negative, which may be related to VBNC forms. 4 77 Sohn et al. have shown that given conducive conditions, the VBNC bacteria within the EBBF matrix can then grow to very high levels in a short period of time. 75

Based on a literature search of clinical studies spanning the past 20 years and evaluating infections related to contaminated endoscopes, the presence of biofilm in endoscope channels was recognized as early as 2004. 79 In this study, the endoscope instrument and air/water channels from 13 different Australian hospitals showed extracellular matrix with embedded bacteria as well as “soil” consisting of lymphocytes, fecal material, and white blood cells. 79 In addition, the need for effective manual cleaning has also been recognized as far back as 2005 80 ; also, the failure of HLD using CIDEX-OPA was demonstrated by Miner et al., who indicated the likelihood of biofilm within colonoscope and gastroscope channels as the basis for 4/4 endoscopes growing >200 CFU of different bacteria. 81 Furthermore, the role of inadequate drying of duodenoscope channels contributing to the outbreak of *K. pneumoniae* (MDRO) was reported by Aumeran et al. 78 Once thorough drying of channels prior to storage had been implemented, the outbreak stopped. 78

These publications demonstrate that as far back as 2010, the key factors related to contamination of flexible endoscope channels was known and included inadequate manual cleaning, accumulation of biofilm over repeated uses in endoscope channels, failure of HLD if biofilm is present in channels, and moisture in channels during storage that facilitates microbial replication leading to biofilm formation. Subsequently, there have been numerous clinical outbreaks of MDROs and investigations supporting these three key components and furthermore showing that contaminated final AER rinse water may also be a source of organisms that subsequently form biofilm within endoscope channels during storage 51 and that using friction as well as neutralizer for sample collection from endoscope channels is needed for successful culture results after HLD. 42 59 77

Persistently, contaminated endoscope channels are not only a potential source of transmission of MDROs to patients (including patients who do not undergo endoscopy procedures but share rooms with those patients who had an endoscopy procedure), but they may also contribute to contamination of the AER final rinse water, healthcare environment, and health care plumbing. 42

There are also several unaddressed clinical parameters that interfere with our ability to control contamination of fully reprocessed patient-used flexible endoscopes. These are summarized in Table 3 but are beyond the scope of this biofilm model review.

**Table 3:** Clinical parameters affecting biofilm formation in endoscope channels.

Issue	Clinical Implications
How to detect established biofilm in endoscope channels; optimal sample collection. For example, do sample-collection methods facilitate biofilm detection (e.g., narrow channels that cannot have flush-brush-flush sample collection)?	False negative cultures allow the continued use of contaminated endoscopes that could transmit pathogens to patients
Can regular rapid cleaning monitoring after manual endoscope cleaning prevent channel biofilm formation?	Poor manual cleaning leads to EFBA that has the potential to transmit pathogens when used on subsequent patients
After hours procedures (i.e., transit time > 60 mins); how to ensure biofilm does not develop (initial biofilm; e.g., 3 h exposure (removable) vs. more mature biofilm 24 h (not readily removable)	Insufficient reprocessing personnel after regular hours leads to prolonged time from endoscope use to full reprocessing. This increases the risk of EFBA formation within endoscope channels
Ensuring friction in narrow channels that cannot be brushed (i.e., flushing detergent only versus providing friction during cleaning stage)	Air/water and auxiliary channels are so narrow that they cannot be manually brushed during manual cleaning. This means there is no friction used in the cleaning process, resulting in EFBA. There is an urgent need for new technology that provides adequate friction for cleaning narrow endoscope channels
Exposure to clinical materials such as; simethicone, lubricants, adhesives, radiographic material, high amount of blood, stool, etc.). What is the impact on cleaning efficacy and biofilm formation?	Use of clinical compounds that interfere with manual and automated cleaning that have not been validated by the endoscope manufacturer; this may facilitate EFBA formation within endoscope channels
Monitoring final rinse water in AERs; there is a lack of MIFU for CFU/mL cutoff and a lack of AER manufacturer instructions for remediation if contamination is found	AERs that are not designed to allow users to easily sample the final rinse water as part of their quality control. This could result in undetected contamination of final rinse water. AER manufacturer instructions are needed for how to remediate the AER if contamination of final rinse water is present
Rapid cleaning verification tests for narrow endoscope channels; do sample-collection methods facilitate EFBA detection?	False negative rapid cleaning tests allow the continued use of improperly cleaned endoscopes. This could lead to undetected EFBA in endoscope channels that could result in pathogen transmission to subsequent patients
Channel inner surface composition/structure; micro-cracks in PTFE – impact on how to optimize friction during channel cleaning	Micro-cracks in the PTFE channel that allow organic and microbial residuals to accumulate and form EFBA in endoscope channels; this could result in pathogen transmission to subsequent patients
Optimal endoscope storage conditions: channel air purging in vertical vs horizontal cabinets (Haak 2025; failure of horizontal storage), 42 storage in channel air purging cabinet vs sealed bag storage	Inadequate documentation required of manufacturers to prove drying adequacy of endoscope channels for horizontal storage modalities. This could lead to biofilm formation in endoscope channels and result in transmission of pathogens to subsequent patients

## Summary

The published data to date confirm that the four key factors leading to EFBA formation in patient-used flexible endoscope channels are: inadequate manual cleaning due to lack of friction and/or shortcuts to the recommended cleaning time, contaminated AER final rinse water, inadequate channel drying during storage, and persistent moisture in channels thereby facilitating biofilm formation/accumulation. Biofilm (specifically EFBA) continues to be a driver of persistent endoscope contamination and exogenous transfer leading to patient infections. This review demonstrates that multispecies EFBA that accumulates in patient-used flexible endoscope channels is very different from other forms of biofilm including HB and DSB. Furthermore, clinical EFBA that is modeled by ECBB or EBBF presents a more realistic challenge that integrates both microbial and organic aspects. These aspects should be integrated into the manufacturer’s validation of flexible endoscope reprocessing MIFU.

It is hoped that endoscope manufacturers choose using a relevant endoscope biofilm channel model to evaluate their instructions for reprocessing and HLD/sterilization. Then, the outcome will provide a significantly better margin of safety for all channels in all reusable flexible endoscopes.

**List of acronyms**
EFBA: Endoscope Fixed-Biofilm Accumulation
DSB: Dry Surface Biofilm
HB: Hydrated Biofilm
HLD: High Level Disinfection
ATCC: American Type Culture Collection
PAA: Peracetic Acid
GLUT: Glutaraldehyde
ARO: Antibiotic Resistant Organism
ERCP: Endoscopic Retrograde Cholangio Pancreatography
AER: Automated Endoscope Reprocessor
ECBB: Endoscope Cyclic Buildup Biofilm
VBNC: Viable But Non Culturable
MDRO: Multi-Drug Resistant Organism
FDA: Food and Drug Administration
HCO: High Concern Organism
MIFU: Manufacturer’s Instructions For Use
LLD: Low Level Disinfection
PTFE: PolyTetraFlouroEthylene
EBBF: Endoscope Buildup Biofilm
RT: Room Temperature
CFU: Colony Forming Unit
WGS: Whole Genome Sequencing
CDC: Centre for Disease Control

## Data Availability

There are no new data provided in this review manuscript.

## Declaration of GenAI Use

The author declares that no Generative AI was used in the preparation of this manuscript.
